# *De novo* assembly of red clover transcriptome based on RNA-Seq data provides insight into drought response, gene discovery and marker identification

**DOI:** 10.1186/1471-2164-15-453

**Published:** 2014-06-09

**Authors:** Steven A Yates, Martin T Swain, Matthew J Hegarty, Igor Chernukin, Matthew Lowe, Gordon G Allison, Tom Ruttink, Michael T Abberton, Glyn Jenkins, Leif Skøt

**Affiliations:** Institute of Biological, Environmental and Rural Sciences, Aberystwyth University, Gogerddan, Aberystwyth, Ceredigion, SY23 3 EB UK; Institute of Biological, Environmental and Rural Sciences, Aberystwyth University, Penglais, Aberystwyth, Ceredigion, SY23 3FL UK; School of Biological Sciences, University of Essex, Wivenhoe Park, Colchester, Essex CO4 3SQ UK; Plant Sciences Unit – Growth and Development, Institute for Agricultural and Fisheries Research (ILVO), Caritasstraat 21, 9090 Melle, Belgium; International Institute of Tropical Agriculture (IITA), PMB 5320, Oyo Road, Ibadan, Nigeria

**Keywords:** Drought stress, Polymorphism, Red clover, RNA-Seq, Transcriptome assembly, Trifolium pratense

## Abstract

**Background:**

Red clover (*Trifolium pratense* L.) is a versatile forage crop legume, which can tolerate a variety of soils and is suitable for silage production for winter feed and for grazing. It is one of the most important forage legumes in temperate livestock agriculture. Its beneficial attributes include ability to fix nitrogen, improve soil and provide protein rich animal feed. It is however, a short-lived perennial providing good biomass yield for two or three years. Improved persistency is thus a major breeding target. Better water-stress tolerance is one of the key factors influencing persistency, but little is known about how red clover tolerates water stress.

**Results:**

Plants from a full sib mapping family were used in a drought experiment, in which the growth rate and relative water content (RWC) identified two pools of ten plants contrasting in their tolerance to drought. Key metabolites were measured and RNA-Seq analysis was carried out on four bulked samples: the two pools sampled before and after drought. Massively parallel sequencing was used to analyse the bulked RNA samples. A *de novo* transcriptome reconstruction based on the RNA-Seq data was made, resulting in 45181 contigs, representing ‘transcript tags’. These transcript tags were annotated with gene ontology (GO) terms. One of the most striking results from the expression analysis was that the drought sensitive plants were characterised by having approximately twice the number of differentially expressed transcript tags than the tolerant plants after drought. This difference was evident in most of the major GO terms. Before onset of drought the sensitive plants overexpressed a number of genes annotated as senescence-related. Furthermore, the concentration of three metabolites, particularly pinitol, but also proline and malate increased in leaves after drought stress.

**Conclusions:**

This *de novo* assembly of a red clover transcriptome from leaf material of droughted and non-droughted plants provides a rich source for gene identification, single nucleotide polymorphisms (SNP) and short sequence repeats (SSR). Comparison of gene expression levels between pools and treatments identified candidate genes for further analysis of the genetic basis of drought tolerance in red clover.

**Electronic supplementary material:**

The online version of this article (doi:10.1186/1471-2164-15-453) contains supplementary material, which is available to authorized users.

## Background

Red clover (*Trifolium pratense* L.) is a versatile forage crop legume, which can tolerate a variety of soils and is suitable for silage production for winter feed and for grazing. The benefits of red clover to farming include nitrogen fixation, soil improvement and high nutritive value in terms of protein-rich feed for livestock. Like other legumes, nitrogen fixation is facilitated by nodulation via symbiosis with the soil microbe *Rhizobium leguminarosum*[1, 2]. The nutritional benefits are attributable to easy digestibility, high voluntary intake by livestock and high protein content during ensiling [3]. Red clover can be grown alone or in a sward mixed with grasses and other legumes, where it has been shown to be more productive than monocultures [4]. Red clover is a short lived perennial that usually persists for two to three years, although more persistent varieties are available. Improving its longevity is a major breeding target [2, 5]. Persistency is a complex trait, and both biotic and abiotic stresses are known to have a major effect on longevity in the field [2]. Red clover has a tap root system in the first year, but in subsequent years a more fibrous root system develops at the expense of the tap root, which senesce [1]. This is believed to increase susceptibility to diseases of the crown, such as crown rot [1]. Abiotic stresses are other factors considered to be of major importance for persistency [1, 3, 5]. Cold and drought stresses are important components of abiotic stress [6–10]. Even temperate forage crops are exposed to periods of drought that have adverse effects on yield and persistency [11]. However, little is known about its genetic basis in red clover. Two studies have identified quantitative trait loci (QTLs) for traits related to persistence in red clover. Using full sib mapping families [12] found a major QTL for all persistency indices measured on linkage group 3, with heritabilites varying between 0.28 and 0.66. Klimenko *et al*. [13] found QTLs related to disease resistence and winter hardiness particularly on linkage groups 3 and 6. These studies are useful for future marker assisted selection. RNA-Seq approaches have potential to provide further valuable information about the molecular mechanisms underlying plant responses to abiotic stresses.

Effects of drought on gene expression have been studied using microarrays in rice [14], wheat [15] and *Arabidopsis thaliana*[16]. These and other studies have provided information about the signalling pathways involved in the response to water stresses [17–19]. A typical response to water stress is to prevent water loss by closing the stomata, and producing abscisic acid (ABA). ABA initiates a signalling cascade which modifies the transcriptome and up-regulates genes encoding a number of proteins and enzymes involved in drought response [20, 21]. These can be divided into signalling and response proteins. Signalling proteins include transcription factors, protein kinases and ubiquitin ligases. Drought response proteins include dehydrins, heat shock proteins, aquaporins and scavengers of reactive oxygen species (ROS) [17, 22–25]. Additionally, metabolic pathways can be modified to compensate for reduced water and CO_2_ uptake [26]. Osmolytes such as proline and pinitol are often produced to lower the osmotic potential in order to maintain water uptake and protect against build-up of toxic ion levels [17].

Next generation sequencing (NGS) can provide a depth of sequencing that is sufficient to cover the transcriptome of an organism many fold and allow quantification of the detected transcripts. The Illumina platform has been used for transcriptome analysis in several plant species [27–32]. An Illumina HiSeq2000 instrument can produce hundreds of millions of paired end reads per flow cell [33–35]. Gene expression is quantified by counting the number of reads per kilobase of transcript per million mapped reads (RPKM), enabling quantitative comparisons to be made [36]. A complication arises with short reads if there is no reference sequence to map the reads onto. In the case of model organisms extensive genomic and transcript sequence data are available, but for non-model organisms the amount of expressed sequence tags (ESTs) and other transcript sequence data available to use as a reference can vary dramatically. Paucity of data in the reference transcriptome can potentially undermine the unbiased potential of NGS transcriptome analysis. Fortunately, software specifically designed for *de novo* reconstruction of the transcriptome from short reads, is available. A variety of methods using short read assemblers have been described in a number of plant species including *Eucalyptus*[30], *Sonneratia alba*[37], chickpea [27], sweet potato [38], alfalfa [28] and lupin [39].

The primary aim of this work was to use NGS technology to study changes in transcriptome patterns in pooled samples of red clover genotypes, contrasting in their phenotypic response to drought. Previous examples of transcriptome analysis have often described effects on seedlings [14, 16, 40] or other shorter term drought treatment. In this work we used mature plants in an experimental set-up designed to mimic field conditions as closely as possible. The plants were F1 progeny of a full sib mapping family. Based on growth rates and relative water content (RWC) we identified two bulks of genotypes contrasting in their response to drought stress. The Illumina platform was used to sequence four pools of RNA samples, two from the drought tolerant bulk before and after drought stress, and two from the sensitive bulk. It enabled us to assemble *de novo* a transcriptome library of red clover from paired end reads. As this study was made on genotypes from a mapping population, it also provided an opportunity to mine the pooled samples for putative SNP and SSR polymorphisms. Finally, information about differentially expressed genes informs our understanding of the fundamental aspects of drought response and identifies potential targets for improved abiotic stress tolerance. As part of this study we have also measured the concentration of a few key metabolites, known from other studies to undergo major changes in concentration in response to water stress.

## Results

The mapping family described in Methods was used to select a subset of genotypes that contrasted phenotypically in their response to drought stress. Leaf material from the two contrasting pools were used as a source for RNA-Seq data to generate a comprehensive library of transcript tags, and to explore how the two pools responded to drought stress in terms of differences in transcript expression patterns.

### Selection of drought tolerant and sensitive genotypes

**Figure 1:**
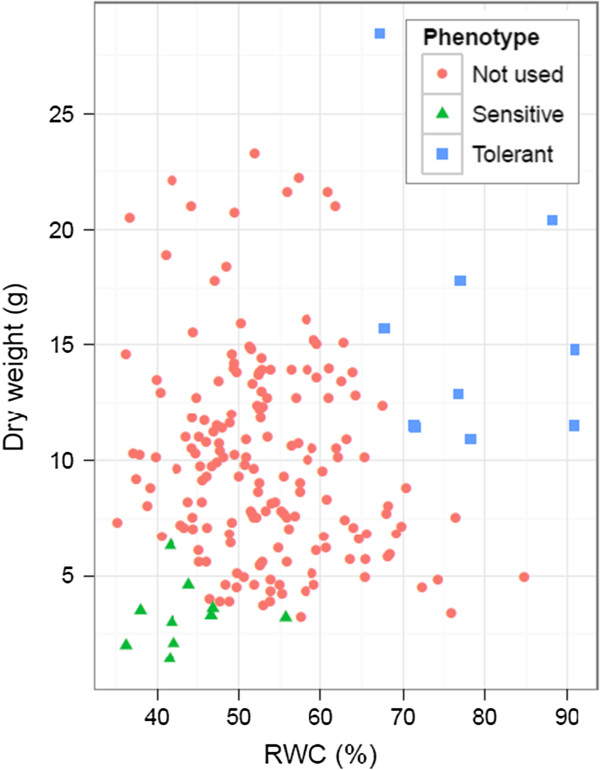
**Scatter plot of plant dry weight against relative water content (RWC) after drought stress.** The ten drought tolerant genotypes are shown as blue squares and the ten sensitive genotypes as green triangles. The remaining genotypes from the mapping family are shown as red circles.

Classification of drought tolerant and sensitive genotypes was based on growth rate (mg dry weight (DW) day^-1^) and RWC at the end of a two month drought treatment (DW2). Drought tolerant genotypes were selected by identifying plants which exhibited the highest RWC and growth rate at DW2. Sensitive genotypes were chosen from plants with the lowest RWC and growth rate. The relationship between the two phenotypic parameters among 64 genotypes from the mapping family is shown in Figure 1, and the ten plants selected from each group are highlighted. Statistical analysis of the phenotypic data from all genotypes measured under control conditions (DW0) revealed no significant differences between the two groups (Table 1A and B). The growth rate was significantly higher in the tolerant pool at the mid drought point (DW1), and at the end of the drought treatment (DW2). The reason for the increased growth rate at mid drought (DW1) in both pools is partly that the soil water content was still 42% compared to 52% at the start of the experiment (Additional file 1), so the plants did not yet suffer appreciably from drought stress. The other reason is the increasing number of daylight hours and increase in average temperature, during the period of the experiment. The continued increase in growth rate of the tolerant plants even at the later stages of the experiment shows their resilience to this stress. This is also illustrated in the difference in RWC and osmotic potential (OP) between the two sets of genotypes at DW2 (Table 1A and C, Additional file 2).

**Table 1:** **Data for plant growth rate, key metabolites, relative water content and osmotic potential**

A				
Sampling times		Sensitive (mg day ^-1^ )	Tolerant (mg day ^-1^ )	***P***
DW0		33 (22-47)	45 (37-54)	*P* = 0.119
DW1		147 (107-204)	286 (239-342)	
DW2		80 (58-109)	336 (269-421)	
DW3		181 (134-244)	369 (302-450)	
**B**				
**Source of variation**		**df**	**MS**	***P***
Pool		1	1.599	<0.001
Covariate		1	0.337	0.003
Residual		17	0.027	
Subject*Time stratum				
Time		2	0.196	<0.001
Time*Pool		2	0.179	<0.001
Residual		36	0.0163	
Total		59		
**C**				
**Trait**		**Sensitive**	**Tolerant**	
RWC	Control	84.2 (81.2-87.2)	85.9 (81.9-89.9)	
	Drought	42.5 (40.0-45.1)	78.0 (72.5-84.5)	
OP	Control	-1.09 (-1.20-(-0.99)) MPa	-1.14 (-1.20-(-1.09)) MPa	
	Drought	-3.60 (-4.14-(-3.06)) MPa	-2.44 (-2.86-(-2.03) MPa	
Glucose	Control	6.94 (5.24-9.19)	6.01 (4.52-7.98)	
	Drought	3.80 (2.60-5.55)	6.74 (5.39-8.42)	
Fructose	Control	7.49 (5.72-9.81)	6.01 (4.57-7.90)	
	Drought	4.77 (3.84-5.94)	9.14 (6.64-12.60)	
myo-Inositol	Control	3.42 (2.76-4.26)	3.29 (2.90-3.74)	
	Drought	2.46 (2.16-2.82)	3.28 (2.79-3.86)	
Malate	Control	14.6 (10.9-19.5)	17.9 (12.8-24.9)	
	Drought	27.6 (20.6-36.9)	22.2 (20.1-24.5)	
Proline	Control	ND	ND	
	Drought	8.51 (6.28-10.74)	5.00 (2.52-7.48)	
Pinitol	Control	17.1 (14.0-20.8)	17.1 (14.3-20.4)	
	Drought	120.9 (110.4-132.3)	109.6 (99.7-120.5)	

A: Plant growth rate and 95% confidence intervals (CI) (in brackets) in the sensitive and tolerant pool. DW0 is growth rate at the onset of the drought stress treatment, DW1 is after 16 days, DW2 is at the end of the drought period (~40 days), and DW3 is growth rate 29 days after the end of the drought stress treatment (recovery). B: ANOVA table for the growth rate data (log_10_ transformed data). The analysis was performed as a repeated measures design with the DW0 (control) as a co-variate. The *P* value next to the DW0 data indicates the significance value for the co-variate only. C: Mean values and 95% CI for relative water content (RWC) (%), osmotic potential (OP), and 6 metabolites mg (gDW)^-1^; ND – below detection limit.

### Red clover transcriptome assembly

The total RNA from both the drought and control treatments for individual plants of the two selected groups was extracted and quality checked. Out of the 40 RNA samples, two failed RNA quality check, one from the sensitive control pool and one from the sensitive drought treatment pool. The RNA samples which passed quality check were then combined in equal amounts in their respective pools and sent to Eurofins Ltd Genomic Service for sequencing. Over 100 M of 100 bp paired end reads were generated after quality checking by Eurofins. *De novo* transcriptome reconstruction of the data was made using RNA-Seq reads and publically available red clover EST sequences. Given the mixed sequencing data, i.e. the short reads produced in this work and the publically available red clover EST sequences (mostly Sanger sequences), this constitutes a hybrid assembly. This approach generated 45181 contigs (denoted ‘transcript tags’ hereafter) constituting 42 Mbp. This Transcriptome Shotgun Assembly project has been deposited at DDBJ/EMBL/GenBank under the accession number GAOU00000000. The version described in this paper is the first one, GAOU01000000. The full list of all transcript tags with their annotation and assignment to GO terms is available in Additional file 3. Other relevant statistics include an average transcript tag length of 933 bp, N50 of 622 bp, 196.4× coverage (actual number of reads mapped/transcriptome length) and longest sequence of 13855 bp. To further evaluate the quality of the assembly, all of the reads were mapped back to the assembled transcriptome using CLC Genomics Workbench v4.0.0. Using a paired end distance of up to 1000 bp 69% of the reads mapped back. A total of 34534 transcript tags (75%) were assigned a functional name by BLAST against Medicago, *Arabidopsis* or UNIPROT, and 29189 transcript tags (63%) were assigned at least one GO term.

### Exploration of gene expression

In total, 6262 transcript tags were differentially expressed (>2 fold) between treatments and pools. The number of transcript tags with increased expression after drought stress were 3546 and 1903, while the number with decreased expression (>2 fold) were 2255 and 1015, in the sensitive and tolerant pool, respectively (Table 2). The full list of differentially expressed transcript tags can be seen in Additional file 4.

**Figure 2:**
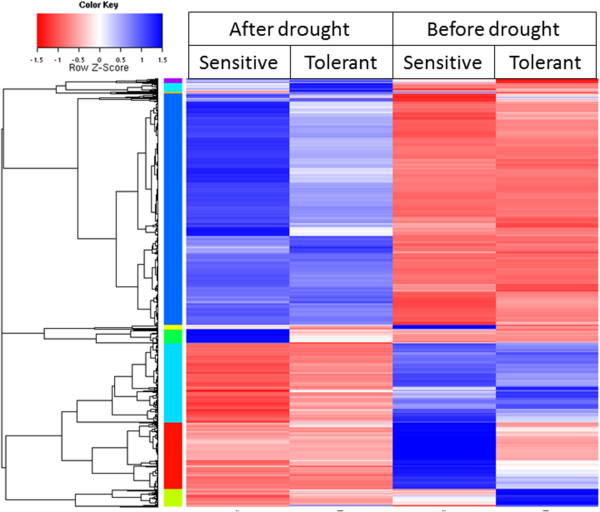
**Clustering analysis of differentially expressed transcript tags.** Heat map of Pearsons correlation across 6350 differentially expressed transcript tags. A dendrogram of correlation between transcript tags is shown to the left of the heatmap.

The results are also summarised in Figure 2, which shows a heatmap (using Pearsons correlation co-efficient). The overall variation in levels of expression was estimated by recording the coefficient of variation (CV) of each identical transcript tag from the sensitive and tolerant samples. The average of the CV for all the pairs of transcript tags was 26.1% before drought (DW0) and 20.2% after drought (DW2) after filtering out tags with < 2 RPKM.

**Table 2:** **Differentially expressed transcript tags in drought stressed red clover leaves compared with control conditions**

Change	Fold difference	Sensitive	Tolerant	Common
Over-expressed	10	526	320	
	5	979	598	
	2	3546	1903	1592
Under-expressed	10	689	137	
	5	1254	322	
	2	2254	1015	851

Results were partitioned between over and under-expressed and the magnitude of difference (fold) in the sensitive and tolerant pools relative to the control.

**Figure 3:**
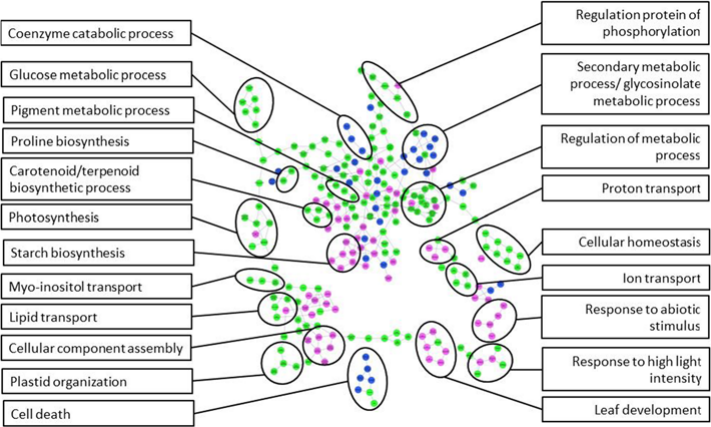
**Map of enriched GO after drought treatment in sensitive and tolerant phenotype pools.** The map shows parent/child connections between GO terms. GO terms are coloured based on significance in two enrichment tests for tolerant and sensitive pools. Green: enriched in both pools, pink: enriched only in sensitive pools and blue: enriched only in tolerant pool. The GO map is annotated with black circles which are linked to summary GO terms, see text for discussion and GO terms.

**Figure 4:**
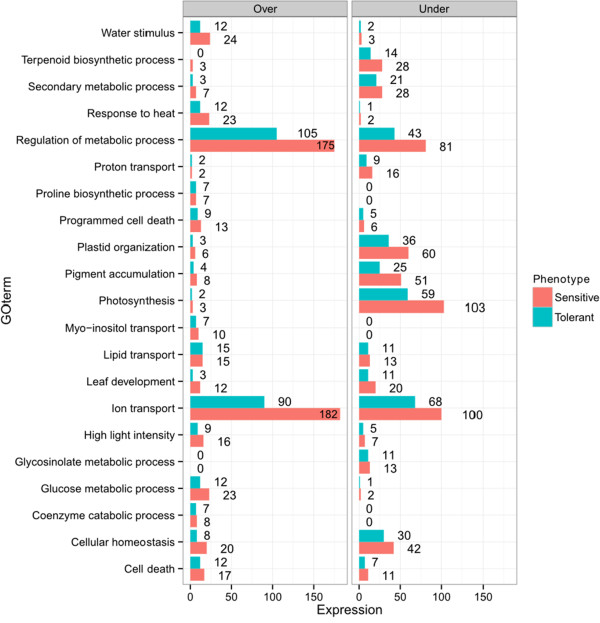
**Number of differentially expressed transcript tags after drought stress.** The data show the number of transcript tags that are over and under-expressed in the tolerant and sensitive pools. The graph shows the GO term on the y-axis and number of differentially expressed transcript tags at the end of each bar, for each GO term.

Analysis of enriched gene ontology terms derived from differentially expressed transcript tags after drought stress revealed GO terms that were common between the drought tolerant and sensitive pools, and GO terms that were unique to each pool. Figure 3 illustrates the GO terms of transcript tags with differential expression. The number of enriched GO terms in common between sensitive and tolerant genotypes was 162, while 38 and 83 unique GO terms were different from the expectation for the tolerant and sensitive pools, respectively. Figure 4 shows the number of transcript tags which were over- and under-represented in various GO terms. The sensitive pool has a larger number of differentially expressed transcript tags in almost all GO term compared to the tolerant pool. Cellular homeostasis (GO:0019725) indicates changes in the steady state physiology of the plants. A total of 20 transcript tags were over-expressed in the sensitive pool compared to 8 in the tolerant pool, and 42 and 30 were under-expressed in the sensitive and tolerant pools, respectively. In the photosynthesis related categories, the following GO terms are highlighted in Figure 3: photosynthesis (GO:0015979), pigment metabolic process (GO:0042440) and plastid organization (GO:0009657). A significant number of transcript tags was down-regulated in all these GO terms. In the metabolism category the highlighted terms represent regulation of metabolic process (GO:0019222), glucose metabolic process (GO:0006006), carotenoid and terpenoid biosynthetic process (GO:0016177, GO:00016114, respectively) and proline biosynthesis process (GO:0006561). Additionally, the following transporter GO terms were found: Myo-inositol, lipid and ion transport (GO:0015798, GO:0006869 and GO:0006810). A number of stress-related GO terms were found in all pools including response to high light intensity (GO:0009644) and cell death (GO:0008219). However, we found that programmed cell death-related terms were specifically enriched relative to expectation in the tolerant pool after drought stress (GO:0012501). Differential expression in response to drought was identified specifically in the tolerant pool for transcript tags relating to GO terms for coenzyme catabolic process, secondary metabolic processes, glycosinolate metabolic processes (GO:0009109, GO:0019748 and GO:0019757, respectively). In the sensitive pool differentially expressed transcript tags related to unique GO terms included proton transport (GO:0015992), starch biosynthesis process (GO:0019252) and leaf development (GO:0048366), as well as a number of abiotic stress GO terms including response to water stimulus (GO:0009415) and response to heat (GO:0009408). Specifically, a transcript tag encoding the key ABA biosynthesis enzyme 9-cis-epoxycarotenoid dioxygenase (NCED1) (RC.21240) was over-expressed in response to drought stress in both pools. Another notable result is that most leucine rich repeat receptor kinases were under-expressed in both pools in response to drought (Additional file 4). These proteins are known to be involved in signal transduction pathways for a range of developmental and defence-related processes, including hormone perception and wound response [41].

While the effect of drought on gene expression was the main focus, we also looked at differences in gene expression between tolerant and sensitive pools under control conditions. The results are shown in Additional file 4 in the ‘control’ tab. The tolerant pool over-expressed 163 (>2 fold) transcript tags compared to the sensitive, which in turn had 436 up-regulated transcript tags. We inspected the data manually for gene functions or pathways and in the tolerant pool we found no consistent patterns in the data and interpret this as natural variation due to transcriptome plasticity. In the sensitive pool we identified eight senescence related proteins (RC.3083, 4561, 7070, 7319, 7433, 10857, 19071, 40938), which were up-regulated compared to the tolerant genotypes prior to drought stress.

### qRT-PCR

**Figure 5:**
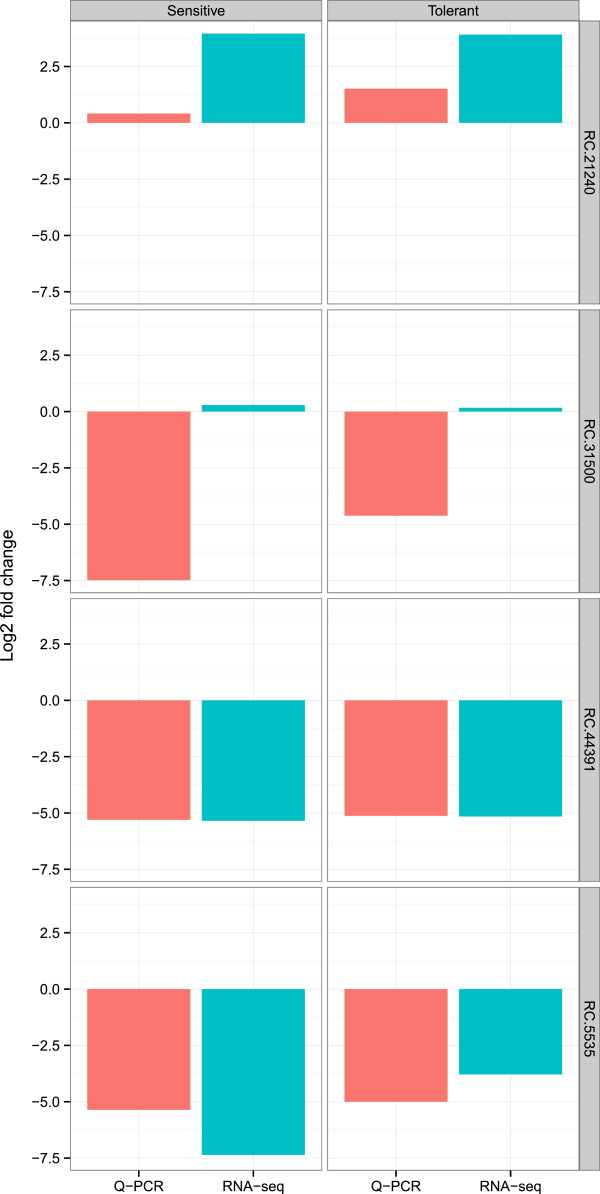
**Expression changes determined by RNA-seq and qRT-PCR in four transcript tags.** The graph shows fold change in expression of four transcript tags (rows) in the sensitive and tolerant pools (columns) as a result of drought stress. Pink refers to qRT-PCR, and blue to RNA-Seq.

To confirm a few of the results from the RNA-Seq work four transcript tags were selected for qRT-PCR analysis. These included three transcript tags, which were differentially expressed (RC.5535 (Matrix metalloprotease), RC.44391 (unknown), and RC.21240 (9-cis-epoxycarotenoid dioxygenase)) and one transcript tag which was expressed constitutively (RC.31500, (ATP synthase delta chain)). The results are shown in Figure 5. For two transcript tags (RC.5535 and RC.44391) the results from the qRT-PCR closely matched the results from the RNA-seq. In the case of RC.21240 the results show a similar trend towards up-regulation, but the fold change in expression is a magnitude higher in the qRT-PCR analysis. The expression level of RC.31500 did not change between treatments in the RNA-Seq experiment, while the qRT-PCR showed a down-regulation as a result of the drought treatment.

### SNP and SSR mining

**Figure 6:**
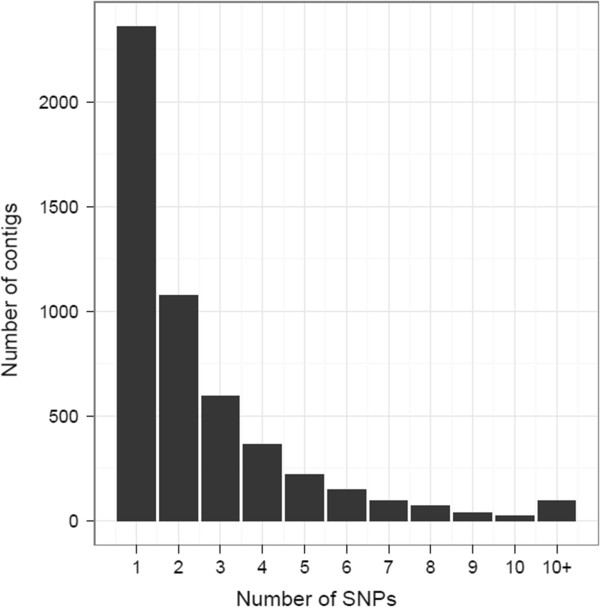
**Number of bi-alleleic SNPs per transcript tag.** Those with more than 10 SNPs are aggregated.

SNP analysis identified 27922 bi-allelic SNP in 7178 transcript tags. This represents on average a SNP per 1.5 kb of transcriptome. The mean distance between SNPs in the 7178 transcript tags containing SNPs was 240 bp. The distribution of SNPs is shown in Figure 6. A total of 462 transcript tags had more than 10 SNPs within them and they accounted for 28% of the total number of SNPs identified. The 10 SNP-richest transcript tags totalled 17429 bp, and contained 519 SNPs, which is about one SNP per 34 bp. The high number of SNPs thus is not due to increased length of the transcript tags. We cannot exclude the possibility that some of them represent paralogous genes, which have been erroneously merged in the assembly process. Transcripts containing single, double or triple SNPs amounted to 2377, 1387 and 859 respectively, accounting for 27.7% of the total number of SNPs. Details of the putative SNPs are shown in Additional file 5. It should be noted that stringent criteria were used to identify these SNPs (see Methods). This means that SNPs were detected in transcript tags with high expression levels. This stringency means that the number of SNPs reported here is likely to be an underestimation of the total number of SNPs in the transcriptome. On the other hand the confidence level in the SNPs listed in Additional file 5 is high. The SNPs will still need to be validated, but their annotation indicate potential polymorphisms in drought and cold regulated transcript tags, as well as ABA responsive elements, and numerous other potential abiotic stress candidate genes (Additional file 5). The fact that the polymorphisms have been identified in genotypes from a full sib family makes genetic mapping of them easier.

The transcript library assembled here also represents a rich source of material for potential transcript-anchored SSR markers. The MISA Perl script (http://pgrc.ipk-gatersleben.de/misa) was used to search for such sequences in the assembly. The results are summarised in Table 2. In total, 3127 SSRs with 2-6 repeat units were identified in 41.9 Mb of transcript sequence data, which amounts to one SSR per 13.42 kb. The number of SSR containing transcripts represented approximately 6% of the total. The largest proportion of SSRs (60%) consisted of tri-nucleotide repeats, with about half as many di-nucleotide repeats. The higher complexity nucleotide repeat SSRs were present in progressively smaller, but still significant, numbers (Table 3). Additional file 6 provides a list of the transcript tags containing SSR motifs, and Additional file 7 is a list of primer pairs designed for amplification of 2193 of the 2744 transcript tags containing SSRs.

**Table 3:** **Breakdown of SSR repeats present in the red clover transcriptome assembly**

SSR data	
Number of seqs searched	45181
Total size of sequences (bp)	41 943 532
Number of SSRs	3127
Number of SSR containing seqs	2744
Number of seqs containing more than one SSR	322
Number of compound SSRs	192
**Distribution of SSR repeat types**	
Di-nucleotides (≥8 repeats)	907 (29%)
Tri-nucleotides (≥6 repeats)	1879 (60%)
Tetra-nucleotides (≥5 repeats)	235 (7%)
Penta-nucleotides (≥5 repeats)	53 (2%)
Hexa-nucleotides (≥5 repeats)	51 (2%)

### Metabolite analysis

The metabolite analysis data summarised in Table 1C show that pinitol, proline and malate all increased in concentration following exposure to drought, while glucose, fructose and myo-inositol remained unchanged. Particularly pinitol concentration rose dramatically from a little over 1.5% of dry matter to over 10% during the course of drought exposure. While proline concentration also rose significantly, it was from a very low base (undetectable), and the overall concentration of this metabolite was much lower than pinitol. Malate concentration was also measured. It can be formed by combining CO_2_ with phosphoenol pyruvate under conditions of limiting CO_2_ availability, such as stomatal closure [42, 43]. In C_4_ plants malate is shuttled between mesophyll and bundle sheath cells, where it is decarboxylated, and recycled. Given that water stress leads to stomatal closure and decreased CO_2_ availability, it seemed reasonable to look for changes in malate concentration in response to drought stress. While the concentration of malate increased (Table 1C), we did not observe convincing evidence for significant changes in expression of transcript tags encoding any of the key enzymes in CO_2_ metabolism which would be necessary for a malate shuttle. This includes phosphoenolpyruvate carboxylase, NADP-dependent malic enzyme, malate dehydrogenase and pyruvate-orthophosphate dikinase.

## Discussion

This work has generated a large amount of sequence data with NGS technology in a non-model crop species. Although red clover previously had a number of resources available, such as an EST database [44] the work described here, has greatly increased the number of transcript sequences available. The high number of sequenced reads resulted in significant coverage of the transcriptome in leaves, allowing gene discovery, quantification of transcripts in four pools and the identification of putative SNPs and SSRs.

In this experiment we used mature plants in a long term (60 days) experiment to simulate the effects of drought as realistically as possible in a greenhouse environment. Many drought experiments described in the literature involving transcriptomics have used seedlings and/or short term drought treatment which minimises variation due to environmental factors (e.g. [14, 16]). While long term experiments are likely to introduce more variation between genotypes and replicate plants, it may also provide potentially novel information on how the plant responds to prolonged periods of water deficiency. A large number of transcript tags were differentially expressed in response to the drought stress in both the sensitive and tolerant plants (Table 2), but variation in expression levels (the average CV of RPKM values of pairs of tags from the sensitive and tolerant pools) was slightly larger before than after drought. We used a pooling strategy to reduce within and between pool differences. This approach has been widely used in RNA-Seq experiments to study for example cell wall composition in Alfalfa [28], berry development in *Vitis viniferia*[29], tissue specific expression in *Eucalyptus*[30] and floral sex determination in cucumber [31]. Barcoding of the four pools enabled multiplexing libraries on a single lane of the flow-cell. This reduced possible confounding effects arising from between-flow-cell differences [45]. It is also interesting to note that expression levels before drought were similar in both pools for 99% of the transcript tags. This suggests that the differences in gene expression which we observed in the two sets of genotypes at the end of the drought stress period are likely to represent a response to the drought stress rather than a difference between the two pools *per se*.

In the qRT-PCR experiment three out of four of the transcript tags tested showed similar changes in expression as was observed in the RNA-Seq experiment. The reasons for the discrepancy between the two methods for RC.31500 are not clear to us. While our data represent a small sample, the results are however of a similar nature to those reported in [29]. They found that 12 out of 15 genes matched the expected RPKM values when quantified using qRT-PCR.

The results reported here represent a platform for further examination of candidate genes with a potential role in plant response to drought stress. The total size of the transcriptome assembled was 42 Mb. This constitutes around 10% of the predicted genome size (440 Mb) [44]. Approximately 46 k transcript tags were generated in the transcriptome reconstruction. This is similar to those found in *Medicago truncatula* (44124 gene loci) [46], Soybean (46430 protein coding genes) [47] and *Arabidopsis* (37019 gene models) [48]. The number and total size of transcripts naturally vary for *de novo* assemblies of other species, such as in chickpea (55 k) transcripts (28 Mb) [27], *Sonneratia alba,* 30 k transcripts (18 Mb) [37] and *Eucalyptus*, 18 k transcripts (22 Mb) [30]. An 8 fold coverage filter on contigs was used in *Eucalyptus,* and a 2× minimum coverage in *S. alba*. This would explain the smaller numbers found in those transcriptomes. Using the same filters for RPKM a similar number of transcript tags would be realised in the present experiment. Due to the heterozygous and allogamous nature of red clover it is likely, that there is some redundancy in our transcriptome assembly. The transcriptome size may thus be over-estimated. On the other hand, erroneously merged assemblies would lead to underestimation of the transcript numbers. The SNP detection process showed that 462 transcript tags had greater than 10 SNPs (Additional file 5), which may suggest that the assembly process in those cases merged paralogues or that the reads could be miss-aligned. The allogamous nature of red clover and the pooling strategy used in the experiment makes it challenging to distinguish with certainty between allelic variants and paralogous genes. Therefore it is reasonable to assume there are a few inconsistencies in the assembled transcriptome. Nevertheless, the majority of the transcript tags do have high coverage and have high similarity to known transcripts.

In this experiment the level of water stress as measured by soil water content was identical across bins at the time of sampling during the drought treatment (Additional file 1). Nevertheless, the sensitive and tolerant plants showed contrasting responses to the same level of soil moisture. The sensitive genotypes had a RWC of approximately 43% (Figure 1). In contrast, the tolerant genotypes had a RWC of 78%, which is only slightly below what is found in non-droughted plants (~85%) (Table 1C). This difference manifested itself by the observation that more than twice the number of transcript tags was differentially expressed in the sensitive pool compared to its control, than was the case in the tolerant pool compared to its control. It would be difficult to infer which differentially expressed transcript tags qualify as genes directly involved in response to the drought stress, and as candidates for targeting in genetic improvement programmes. Nevertheless, the results do provide a comprehensive overview of gene expression changes after drought stress. The number of differentially expressed transcript tags which were detected as a result of the drought treatment (Additional file 4) are similar to those reported in other species such as rice (3097 up, 2391 down) [14], wheat (3056 differential) [15] and *Arabidopsis* (2059 up and 2075 down) [16], albeit for shorter term drought stress.

There was a large degree of overlap between GO terms of differentially expressed transcript tags between the sensitive and tolerant pools in their response to drought. The main difference is that a larger number of transcript tags from the sensitive pool with the same GO terms were either up-regulated or down-regulated in response to the drought, compared to the tolerant pool (Figure 4). The reason for this is not clear, but could simply be a consequence of the degree of severity of the stress experienced by the plants in the two pools. Down-regulation of the photosynthetic apparatus also involves more genes in the sensitive pool than the tolerant, possibly also caused by the difference in sensitivity to the stress (Figure 4). The results contrast with those of [49], who found that more genes were up-regulated in tolerant barley genotypes than in sensitive. Comparisons with short term drought experiments are however, difficult to make. The gradual onset of drought conditions in the present experiment may elicit the differential expression of more genes to counteract the severity of the stress. Nevertheless, even the relatively modest reduction of ~10% in RWC observed in the tolerant pool, elicited a significant transcriptome response albeit after prolonged water limitation.

Another finding is the high number of senescence-related transcript tags that were up-regulated in the sensitive pool in non-droughted plants. Perhaps the sensitive plants have a predisposition for reduced growth and less stress tolerance [50]. Chaves *et al.*[19] suggested that plants not predisposed to early senescence may be of value for breeding purposes. Identification of molecular markers or other biomarkers associated with these differences could potentially assist in the removal of such sensitive plants from the gene-pool in a population-based breeding programme. In two of those transcripts (RC.7319 and RC.19071) SNPs were identified (Additional file 5).

The transcript tags which were differentially expressed are similar to those from other drought studies where large transcriptome profiling was used [14–16]. They can be divided into three main groups: functional, regulatory and photosynthesis related genes. Functional drought response genes include protection enzymes (LEA, heat shock proteins, ROS scavenging), osmolyte biosynthesis (proline, trehalose, pinitol/ononitol), transporters (aquaporins, sugar transporters). Regulatory proteins include transcription factors (MYB, NAC, DREB and zinc finger), and those involved in post-transcriptional modification (splice factors, DEAD-box) and post-translational modification, (ubiquitin ligases, protein kinases) and epigenetic factors (histones) [15–17]. When plants are unable to use or dissipate absorbed light energy, the excess energy leads to production of ROS which can cause oxidative damage to the photosynthetic machinery [19]. Under prolonged drought stress, the photosynthetic machinery is down-regulated [51]. This is also the case in this study, where particularly transcript tags encoding Rubisco and polypeptides involved in PSII and PSI were down-regulated (Additional file 4). The rate of recovery of the plant after drought is tightly linked to photosynthetic recovery [18]. Therefore plants which maintain larger portions of their photosynthesising apparatus would have an advantage over those with a lower portion once the stress is alleviated.

Three metabolites known to accumulate in plants in response to drought were measured in this work. Pinitol accumulation was most dramatic and statistically significant (Table 1C). We therefore tried to identify a candidate gene likely to represent the O-methyl transferase activity required for the key step in the biosynthesis of pinitol from myo-inositol [52]. The greatest homology with the orthologue in the ice-plant (*Mesembryanthemum crystallinum*) was RC.4122, but when we did a BLAST search with that as the query, most of the best hits were to genes annotated as caffeic acid-O-methyl transferase. Furthermore, expression of RC.4122 did not increase in response to drought. One explanation could be that the accumulation of pinitol took place early in the drought stress period, and expression of the gene had declined by the end of the drought period. That seems unlikely, since transcript tags annotated as two of the key enzymes in proline biosynthesis were over-expressed in both the sensitive and the tolerant pool in response to drought (e.g. RC.34920: pyrroline-5-carboxylate reductase (P ≤ 7.99 × 10^-5^), and RC.35692: Δ^1^-pyrroline-5-carboxylate synthetase (P < 10^-200^) (Additional file 4). However, the similarity between most O-methyl transferases makes accurate annotation more challenging. It is surprising to us though, that this gene does not appear to have been identified in any legume despite the high concentration of pinitol and other polyols in this family [53–55].

## Conclusions

This work makes available the first transcriptome library of red clover using next generation sequencing technology. The library was constructed using two pairs of pools of ten genotypes from an F_1_ mapping family. RNA from the two pairs of pools (drought sensitive and drought tolerant, as determined by phenotypic analysis) was extracted before and at the end of a 60 day period of drought. The RNA-Seq analysis provided a total of 45181 transcripts longer than 200 bp. A large number of transcript tags involved in the photosynthetic apparatus were down-regulated in both the sensitive and tolerant pools, but particularly in the former. A great many transcription factors, genes involved in protein modification and degradation were up-regulated in both pools after drought, but again, particularly in the sensitive pool. As expected, a number of genes involved in ABA biosynthesis and abiotic stress signal transduction were also up-regulated. The general trend was that a larger number of genes were up-regulated in the sensitive pool than in the tolerant. Despite the highly significant increase in the concentration of the compatible solute pinitol, we were unable to definitively identify the gene encoding the O-methyl transferase responsible for the key step in pinitol biosynthesis from myo-inositol. We recognise the limitations of the quantitative aspects of this work, and that further validation of the assembly is needed, but the results presented here provide a valuable resource for future work in terms of SNP identification, and annotation of genome sequence assembly currently in progress.

## Methods

### Plant material and experimental setup

The mapping family used throughout consisted of progeny from a F_1_ pseudo-testcross derived from a single genotype from each of two varieties, Milvus and Britta. The plant material was generated initially using a clonal cutting method from the original plant material involving excision of stem regions with nodes followed by auxin treatment and rooting in soil. Subsequent clones were generated by clonal splitting [56]. We made use of 64 genotypes from the mapping family. Three blocks were used in the experimental design, where each block constituted one clone of each of the 64 genotypes. Each block consisted of two drought bins in a randomised block design, i.e. 32 plants per bin and 64 plants per block. The ‘drought bins’ measured 80 cm by 110 cm, with a depth of 80 cm. The bins were filled with gravel up to 30 cm: the remaining 50 cm was filled with John Innes Number 3 compost. Prior to the start of the experiment the bins were watered to field capacity.

Plant material was grown to mature size in 15 cm pots before transfer to drought bins on 06/03/2010. Plants were then allowed to adjust 2 days to the new environment before cutting back to approximately 6 cm height above soil level. Then the bins were watered daily to maintain field capacity (~55% soil moisture content) until the plants reached a mature size on 15/04/2010, at which time they were sampled for multiple phenotypic traits as described below. This was designated as DW0. From then onwards all watering ceased. Plants were cut again on 05/05/2010, and dry weight was recorded. This was designated as DW1. The subsequent sampling time for individual bins (designated DW2) was done when soil moisture content was down to 14% (see next section for details). This happened between 13-18/06/2010. All phenotypic traits were sampled at this point. Subsequently, the bins were fully hydrated and continually watered until the last cutting on 16/07/2010. This was designated DW3. Growth rate was quantified as dry weight divided by the number of days of growth since the previous cut.

### Soil moisture, relative water content and osmotic potential

Two access tubes for Delta-T PR1 Profile probes were inserted into each drought bin. They were placed equidistant from the edge and 30 cm apart. The soil moisture content was measured using Delta-T PR1 Profile probe and Delta-T HH2 moisture meter at soil depths of 10, 20, 30 and 40 cm. The mean of the 4 measurements was used to indicate the water content of the soil in the bin. In addition, five plants per bin were sampled randomly at 8:30 am for measurement of relative water content (RWC). A single leaf sample was excised from each plant and placed in a sealable zip-lock bag on ice before quantifying RWC using the method described by Smart and Bingham [57]. The soil moisture content and RWC were used as indicators of the drought status of the bins and plants, respectively. The final sampling was done when the soil moisture average fell below 14% and/or the RWC was below 60%.

Samples for measurement of osmotic potential (OP) were collected between 15:00 and 16:00, by excising mature leaf material and storing at -20°C. OP was then quantified using a Wescor vapour pressure osmometer 5520 (Wescor Inc., USA) as described by the manufacturer.

### RNA sampling

Samples for RNA extraction were collected between 13:00 and 15:00. The youngest fully open trifoliate leaf was used for this. The excised material was stored in 2 ml safe-lock Eppendorf tubes and then flash frozen in liquid N, before storing at -80°C. The leaf sample was ground to a fine powder using a Retsch MM300 mixer mill. RNA was then extracted using the Trizol (Invitrogen, USA) method as described by the manufacturer, with an additional chloroform extraction step added. Total RNA was solubilised in 50 μl DEPC-treated H_2_O. A 2 μl aliquot was taken and diluted 1:5 in DEPC treated water and used for RNA quantification. Quantification was done using BioRad Experion Automated Electrophoresis System with RNA StdSens chips (Bio-RAD Laboratories Inc., USA), as described by the manufacturer using 3 μl of the diluted RNA extract. The data were analysed using Experion™ Software System operation and data analysis tools, version 3.2.243.0 copyright © 2010 Bio-Rad laboratories. Four pooled samples were generated (see Results section), two consisting of 9-10 drought sensitive genotypes sampled before and at the end of the drought treatment, and two consisting of 9-10 drought tolerant genotypes exposed to the same treatments. For each pool, 2 μg of total RNA from each sample was included. Then 500 μl of 100% ethanol was added to each pool, and kept at -20°C during transport.

### Sequencing and transcriptome construction

The four RNA pools were sent to Eurofins Genetic Services (Eurofins, Germany), for sequencing. This included preparation of 4 barcoded libraries, which were multiplexed on a single flow-cell of an Illumina HiSeq2000 (Illumina, USA). The sequencing was done as paired-end reads, 2×100 bp in length. The data was quality checked at Eurofins and provided as four libraries in FASTq format. Sequence quality controls were applied by Eurofins and the read libraries were checked using fastqc (http://www.bioinformatics.babraham.ac.uk/projects/fastqc/). After assembly the contigs were checked for adaptor sequences and none were found.

For the initial construction of the reference transcriptome Velvet [58] was used. Assemblies were created using k-mer sizes of 31, 41, 51 and 61. The 61-k-mer length assembly was chosen for further use because it gave the largest N50, the largest average scaffold size, the largest size of any single scaffold, and the smallest number of scaffolds. This generated 93.4 k contigs. To increase the length of contigs and reduce the number of redundant contigs the Velvet assemblies were combined with existing sequences from the databases from the publically available *Trifolium pratense* EST library, downloaded from http://www.plantgdb.org/download/download.php?dir=/Sequence/ESTcontig. The red clover ESTs were combined with the Velvet assemblies by using a second *de novo* assembly on CLCbio Genomics Workbench version 4.0.0 (31 k-mer). This created 47229 contigs of at least 200 bases in length. In order to retain polymorphism data, conflicting bases found during the assembly process were called ‘ambiguously’, rather than using the ‘vote’ option. Next, bacterial contigs were removed, by compiling over 4000 bacterial genomes from the NCBI RefSeq database. Then the transcriptome contigs were mapped against the bacterial genomes and un-mapped (non-bacterial) contigs were retained. This led to the removal of 634 contigs. However, to remove the ambiguity for submission to NCBI the reads were mapped back to the contigs to give a ‘vote’ scoring.

Transcript tag expression was determined using the method described by [36] and executed in CLC. For identification of differentially expressed transcript tags statistical analysis described by [59] was used. A false discovery rate (FDR) of 0.05 was used as the threshold for significance.

### Annotation and gene ontology analysis

BLASTX was used with minimum E-value 1e-15 for annotation of transcript tags. Sequentially, contigs were searched using BLASTX against the following protein databases *Medicago truncatula* (v3.5, [46]), *Arabidopsis thaliana* (TAIR10, [48]) and the Universal Protein Resource (UniProt, [60, 61]). From each BLAST output the best match was used to annotate the transcript tags. After each BLAST search annotation tags with no matches and ones with ‘hypothetical’, ‘predicted’ or ‘unknown’ annotations were extracted for next sequential BLAST search (order: Medicago - *Arabidopsis* – UniProt). For GO assignment corresponding GO terms were downloaded from relevant databases. A small number of tags annotated as fungal were removed by manual curation.

TopGO from Bioconductor in R (http://www.r-project.org/) was used to identify enriched GO terms. This was done for all differentially expressed genes, split between over- and under-expressed transcript tags identified by CLC, for each pairwise comparison. The Fishers test implemented in TopGO was used to identify enriched GO terms relative to its expectation. GO terms with a *P*-value < 0.01 were selected. Both common and unique GO terms were identified by intersection of the data, including those unique to individual pools. Cytoscape [62] was used to visualise the selected GO terms. The GO map for *A. thaliana* was downloaded and installed in Cytoscape, the enriched GO terms were selected and a sub-network was created for visualisation of the GO network.

### qRT-PCR

Aliquots of the RNA extracts were DNAse treated with the Ambion DNA-Free™ kit (Ambion, USA), according to the manufacturer instructions. The cDNA synthesis was done using Invitrogen™ Superscript™ II reverse transcriptase (RT) (Invitrogen, USA) as described by the manufacturer using an Oligo(dT)^25^ Primer with 50 ng total RNA. Samples were then treated with RNaseH (Promega, USA) and stored at -20°C. All qRT-PCR work was done using an Applied Biosystems (USA) 7500 Real time PCR system. The 7500 system sequence detection software version 1.2.3 was used for analysis including auto Ct/CP (cross threshold/crossing point), mean CP and outlier removal at 95% confidence). All samples were run in triplicate using the following PCR concentrations in a volume of 10 μl: 5 μl SYBR Green master mix (Applied Biosystems, USA), 900nM forward and reverse primers (Biolegio, Netherlands), and 1 μl 10× diluted cDNA. All primers were designed using the Roche Universal ProbeLibrary Assay Design Center at https://www.roche-applied-science.com/sis/rtpcr/upl/index.jsp?id=UP030000. The following amplification program was used: 95°C for 10 min, 40 cycles at 92°C for 15 s, annealing temperature for 30s, 72°C for 45 s followed by a final elongation at 72°C for 10 min. The specific annealing temperatures and nucleotide sequence of the primers are shown in Additional file 8. The relative expression software tool (REST-384) was used to calculate the relative fold change in gene expression (http://www.gene-quantification.info/) [63] using the mathematical model described by Pfaffl [64]. Primer efficiencies were measured using a serial dilution of stock cDNA at 1:1, 1:10 and 1:100. Every plate included three negative controls for detection of contamination. RC.44146 was used as the reference gene. This was the Actin gene, which was found not to be differentially expressed in the RNA-seq data analysis. For comparison between RNA-seq and qRT-PCR data, the fold change in expression of drought stressed samples was calculated relative to control samples for both RNA-seq and qRT-PCR data.

### SNP and SSR mining

The SNP detection tool in CLC was used to identify SNPs. All reads were included in this process. The minimum count for the variant allele was 200 and window length was 17 bp. For identification of SNPs inherited from one heterozygous parent and one homozygous parent, the minimum variant frequency was set to 20% to allow variation from the expected haplotype frequency of 25/75%.

The MISA (Microsatellite) Perl script (http://pgrc.ipk-gatersleben.de/misa) was used for identification of SSRs. The settings for minimum number of repeats were as follows: Di-nucleotide 8; Tri-nucleotide 6; and Tetra, Penta and Hexa-nucleotide repeats 5. For compound SSRs the maximum distance between two SSR runs was 50 bp. The BatchPrimer3 programme [65] was used to design primers pairs for amplification of the SSR motifs. The default settings were used except for the annealing temperature, which was set for an optimum of 60°C.

### Metabolite sampling and extraction

Whole young trifoliate leaf samples were collected for metabolite quantification between 11:00 and 13:30. They were stored on ice in re-sealable plastic bags, until an amount of 20-60 mg was weighed. This was stored in 2 ml safe-lock microfuge tubes (Eppendorf, Germany) before they were flash frozen in liquid N_2_ and then stored at -20°C. Before extraction a metal ball bearing was added to each tube. During this process the samples were kept frozen by brief exposures to liquid N_2_. This was also the case when the samples were homogenized to a fine powder in a Retsch-mill MM300, mixer mill (Retch, Germany) for 2 min (20 impacts/s). Afterwards 300 μl of 100% ethanol was added and mixed for a further 15 minutes (20 impacts/s). Next, 200 μl of chloroform (CHCl_3_) was added and mixing continued for 5 minute (20 impacts/s). Then, 400 μl H_2_O was added and the samples were mixed by vortexing, before centrifugation for 5 min at 14 k rpm to separate the aqueous ethanol phase from the chloroform phase. The total volume of the aquous ethanol phase was approximately 525 μl. Two 75 μl-aliquots were transferred to new microfuge tubes (1.5 ml). The aliquots were dried using a speed vac concentrator for four hours (without heating), with the lids open under vacuum. All samples were then stored at 4°C.

### Determination of metabolite concentration using gas chromatography mass spectroscopy

Prior to derivatization of the samples two internal standards were added: castanospermine (Enzo life sciences, USA) (20 mg) and cyclo-leucine (Sigma-Aldrich, UK) (20 mg). The samples were dried down completely before derivatizing. Derivatization of the samples was achieved using a 2-step process. First, 30 μl of freshly prepared MOX solution (O-methylhydroxylamine hydrochloride in pyridine, Sigma-Aldrich, UK) was added to the dried samples, and kept at room temperature for 2 hours to allow solubilisation. The samples were then transferred to crimp sealed 0.3 ml GC vials (Chromacol/Fisher, UK), and the vials were heated for 15 minutes at 90°C to convert ketone groups to the oxime derivative. The vials were then de-capped and 20 μl BSTFA (N,O-Bis(trimethylsilyl) trifluoroacetamide) (Sigma-Aldrich, UK) was added. The vials were recapped and heated for a further 15 minutes at 90°C to convert remaining polar groups to their trimethylsilyl derivatives. This method is similar to those described by Parveen *et al.*[66].

Analysis was carried out using an Agilent GC-MS system (Agilent Technologies UK Ltd.) comprising a 5973 network Mass Selective detector, an 6890 Series GC and a 7683 series autosampler. The GC was fitted with a Varian FactorFour™ VF-5 ms capillary column (30 m × 0.25 mm ID × 0.25 μm film thickness). The injection volume was set to 1 μl with a 1:50 split ratio. The oven was set at an initial temperature of 80°C and increased to 280°C at a rate of 10°C per minute. The inlet temperature was set to 280°C and the transfer line to 320°C. The mass spectrometer was set to scan between 50 and 600 m/z. For quantification of metabolites standard curves were prepared using standards (Sigma-Aldrich, UK with the exception of pinitol which was purchased from ACROS Organics, Belgium). Quantification was achieved using Enhanced Data Analysis software (ChemStation 1701 CA version 00.00, Agilent Technologies UK Ltd.). Additional file 9 shows the metabolites that were quantified and relevant information for quantification. The quantification of glucose, fructose, *myo-*inositol and pinitol in non-droughted samples required standard curves ranging from 1-50 μg. In droughted samples the higher concentrations of pinitol required the range to be increased to 500 μg. An additional standard curve was set up for pinitol concentrations above 200 μg since the curve was non-linear in the high range.

### Statistical analysis of plant growth and metabolites

The plant growth data were analysed using a repeated measures ANOVA approach with the control data (DW0) as a co-variate. The data were log_10_ transformed. Because there were only two timepoints for the RWC, OP and metabolite data, we used a split-plot in time ANOVA design for the analysis of those data. The data for glucose, fructose, myo-inositol, malate and pinitol were log_10_ transformed. The analysis was performed using Genstat, 15^th^ edition (VSN International Ltd (http://www.vsni.co.uk)).

## Availability of supporting data

All the sequencing data have been deposited in the NCBI short read archive under the Bioproject PRJNA219226 (http://www.ncbi.nlm.nih.gov/bioproject/PRJNA219226). Other supporting data are included as additional files.

## Electronic supplementary material

Additional file 1: **Figure illustrating the change in soil moisture content (%) during the drought experiment.** The vertical red lines represent key time points in the course of the experiment. From left to right: DW0 – onset of drought; DW1 – mid-drought; DW2 – end of the drought treatment. (PDF 10 KB)

Additional file 2: **Analysis of variance (ANOVA) table for the trait data presented in Table** 1 **C.** The analysis was performed as a split-plot in time design. The data for glucose, fructose, myo-inositol, malate and pinitol were log_10_ transformed. The analysis was performed using Genstat 15^th^ edition (VSN International Ltd; http://www.vsni.co.uk). (DOCX 17 KB)

Additional file 3: **Annotation of the 45181 red clover transcript tags.** Excel file showing the annotation of the red clover transcript tags as well as a description of the GO terms. (XLSX 2 MB)

Additional file 4: **Differentially expressed transcript tags in the sensitive and tolerant pools.** The file is separated into three sheets: sensitive, tolerant and control for respective pairwise comparisons. The sensitive and tolerant tabs show differential expression in the two pools in response to drought stress. The control tab shows differentially expressed transcript tags from pairwise comparison of tolerant and sensitive pool before drought stress. The table shows relevant statistics and functional description in addition to expression values. (XLSX 1 MB)

Additional file 5: **Transcript tags containing SNPs.** The excel file shows the SNP polymorphism, their annotation, the position of the SNP in the transcript tag and read counts of each allele. (XLSX 1 MB)

Additional file 6: **Red clover transcript tags with SSR motifs.** The SSRs were identified according to the Perl MISA script (see Methods), and the file lists the characteristics of the SSRs. (XLSX 146 KB)

Additional file 7: **Primer design for amplification of SSRs.** This file provides information about primer design for the amplification of 2139 of the 3127 SSR motifs identified. (XLSX 450 KB)

Additional file 8: **Primers for qRT-PCR experiment.** (DOCX 15 KB)

Additional file 9: **Relevant parameters for the gas chromatography mass spectroscopy (GC-MS) analysis.** (DOCX 16 KB)

## Abbreviations

BLAST: Basic local alignment search tool
bp: Base pair
DREB: Dehydration responsive element binding
LEA: Late embryogenesis abundant
NADP: Nicotinamide adenine dinucleoside phosphate
PSI & PSII: Photosystem I & II
qRT-PCR: Quantitative real time polymerase chain reaction
ROS: Reactive oxygen species
Rubisco: Ribulose bisphosphate carboxylase
TAIR: The Arabidopsis Information Resource.
