# Supplementary figures and images for: De novo assembly of red clover transcriptome based on RNA-Seq data provides insight into drought response, gene discovery and marker identification
Source: BMC Genomics. 2014 Jun 9;15(1):453. doi: 10.1186/1471-2164-15-453 (PMC4144119; doi:10.1186/1471-2164-15-453)

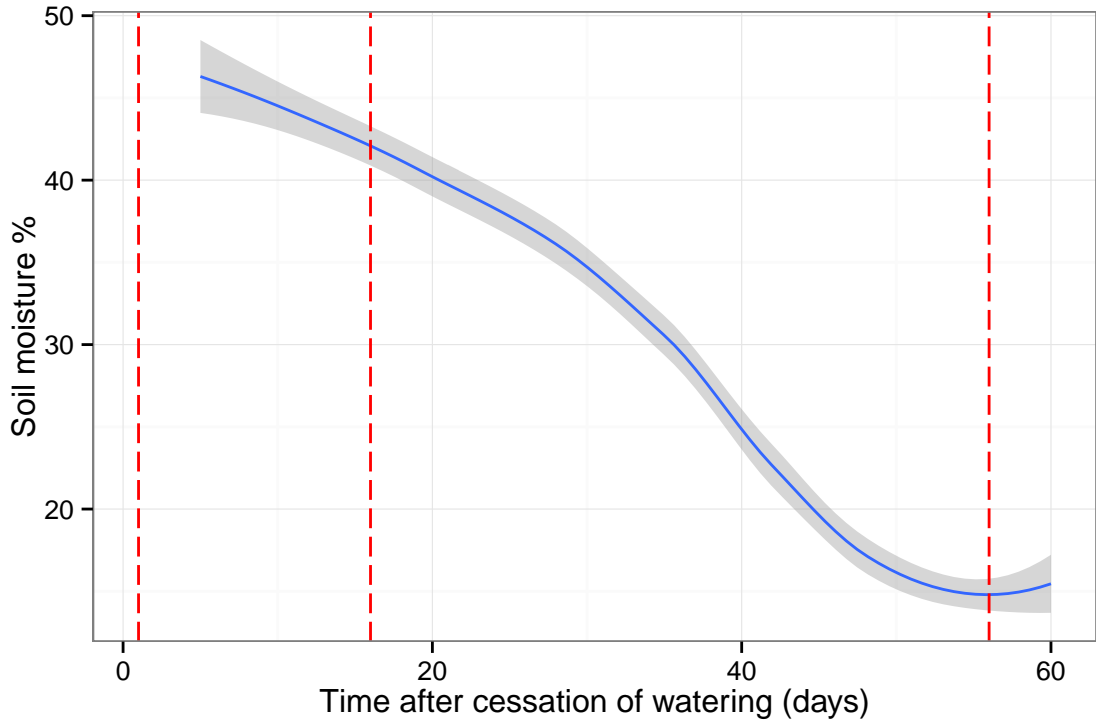

Supplement: Supplementary file 1 — Additional file 1: Figure illustrating the change in soil moisture content (%) during the drought experiment. The vertical red lines represent key time points in the course of the experiment. From left to right: DW0 – onset of drought; DW1 – mid-drought; DW2 – end of the drought treatment. (PDF 10 KB) [file 12864_2013_6182_MOESM1_ESM.pdf]
