# Supplementary material for: De novo assembly of red clover transcriptome based on RNA-Seq data provides insight into drought response, gene discovery and marker identification
Source: BMC Genomics. 2014 Jun 9;15(1):453. doi: 10.1186/1471-2164-15-453 (PMC4144119; doi:10.1186/1471-2164-15-453)
Supplement: Supplementary file 2 — Additional file 2: Analysis of variance (ANOVA) table for the trait data presented in Table 1 C. The analysis was performed as a split-plot in time design. The data for glucose, fructose, myo-inositol, malate and pinitol were log10 transformed. The analysis was performed using Genstat 15th edition (VSN International Ltd; http://www.vsni.co.uk). (DOCX 17 KB) [file 12864_2013_6182_MOESM2_ESM.docx]

| **Additional file 2. Analysis of variance (ANOVA) table for the trait data presented in Table 1C.** The analysis was performed as a split-plot in time design. The data for glucose, fructose, myo-inositol, malate and pinitol were log_10_ transformed. The analysis was performed using Genstat v 15 (VSN International Ltd; http://www.vsni.co.uk). | | | |
| --- | --- | --- | --- |
| **RWC** | | | |
| Source of variation | df | MS | *P* |
| Pool | 1 | 3458 | 1.44e-08*** |
| Residuals | 18 | 37 |  |
| Within | | | |
| Time | 1 | 6165 | 1.12e-10*** |
| Pool:Time | 1 | 2846 | 4.83e-08*** |
| Residuals | 18 | 36 |  |
| **OP** | | | |
| Source of variation | df | MS | *P* |
| Pool | 1 | 3.0800 | 0.0014** |
| Residuals | 18 | 0.2165 |  |
| Within | | | |
| Time | 1 | 36.29 | 4.35e-10*** |
| Pool:Time | 1 | 3.63 | 0.00123 |
| Residuals | 18 | 0.25 |  |
| **Glucose** | | | |
| Source of variation | df | MS | *P* |
| Pool | 1 | 0.0866 | 0.0763^ns^ |
| Residuals | 18 | 0.0245 |  |
| Within | | | |
| Time | 1 | 0.1118 | 0.0563^ns^ |
| Pool:Time | 1 | 0.2422 | 0.0077** |
| Residuals | 18 | 0.0268 |  |
| **Fructose** | | | |
| Source of variation | df | MS | *P* |
| Pool | 1 | 0.0867 | 0.08^ns^ |
| Residuals | 18 | 0.0252 |  |
| Within | | | |
| Time | 1 | 0.0004 | 0.9007^ns^ |
| Pool:Time | 1 | 0.3576 | 0.0021** |
| Residuals | 18 | 0.0276 |  |
| **myo-Inositol** | | | |
| Source of variation | df | MS | *P* |
| Pool | 1 | 0.0280 | 0.119^ns^ |
| Residuals | 18 | 0.0104 |  |
| Within | | | |
| Time | 1 | 0.0519 | 0.0233* |
| Pool:Time | 1 | 0.0505 | 0.0249* |
| Residuals | 18 | 0.0084 |  |
| **Malate** | | | |
| Source of variation | df | MS | *P* |
| Pool | 1 | 0.0002 | 0.939^ns^ |
| Residuals | 18 | 0.0257 |  |
| Within | | | |
| Time | 1 | 0.3427 | 0.0015** |
| Pool:Time | 1 | 0.0833 | 0.0817^ns^ |
| Residuals | 18 | 0.0245 |  |
| **Pinitol** | | | |
| Source of variation | df | MS | *P* |
| Pool | 1 | 0.0043 | 0.443^ns^ |
| Residuals | 18 | 0.0071 |  |
| Within | | | |
| Time | 1 | 6.865 | 3.05e-16*** |
| Pool:Time | 1 | 0.005 | 0.473 |
| Residuals | 18 | 0.009 |  |

.
