# Supplementary material for: De novo assembly of red clover transcriptome based on RNA-Seq data provides insight into drought response, gene discovery and marker identification
Source: BMC Genomics. 2014 Jun 9;15(1):453. doi: 10.1186/1471-2164-15-453 (PMC4144119; doi:10.1186/1471-2164-15-453)
Supplement: Supplementary file 8 — Additional file 8: Primers for qRT-PCR experiment. (DOCX 15 KB) [file 12864_2013_6182_MOESM8_ESM.docx]

| **Additional file 8.** **Primers for quantitative PCR.** Contig ID, annealing temperature for PCR cycling, the primer orientation and the sequence (5’-3’ orientation) is displayed. | | | |
| --- | --- | --- | --- |
| Contig | Annealing  temp (°C) | Primer orientation | Sequence |
| RC.44146 | 55 | forward | TTAATGTGCCTGCCATGTATGT |
|  |  | reverse | GACCACTTGCATAAAGGGAGA |
| RC.5538 | 60 | forward | CTAGGGTTGGGGCATTCTC |
|  |  | reverse | TTTTTGGTAACATAATCGGATACATAA |
| RC.44391 | 60 | forward | TGCTGCAGTTATTGTTTTTGGA |
|  |  | reverse | AAAATTACAAAATTCCGGCTGTT |
| RC.31500 | 60 | forward | TGATGAAACCAACACAACTTGA |
|  |  | reverse | GTTGGAAGAGTTCGTGAGGATT |
| RC.21240 | 55 | forward | TCGACGAGGAAAGCGATAAT |
|  |  | reverse | TTCTACCCAATTTGTTCTTGTTCA |
