# Supplementary material for: De novo assembly of red clover transcriptome based on RNA-Seq data provides insight into drought response, gene discovery and marker identification
Source: BMC Genomics. 2014 Jun 9;15(1):453. doi: 10.1186/1471-2164-15-453 (PMC4144119; doi:10.1186/1471-2164-15-453)
Supplement: Supplementary file 9 — Additional file 9: Relevant parameters for the gas chromatography mass spectroscopy (GC-MS) analysis. (DOCX 16 KB) [file 12864_2013_6182_MOESM9_ESM.docx]

**Additional file 9.** **Metabolite quantification parameters for GC-MS analysis.** The ‘internal standard reference’, refers to which internal standard was used for quantification. Target Ion, the MS peak used for quantification and Q1 (qualifier) Ion is the second peak for MS quantification. Relative response refers to the ratio between target Ion and Q1 ion. R^2^ is the regression coefficient for the standard curve using Enhanced data analysis software. * = internal standard.

| **Metabolite** | **Internal standard reference** | **Retention time (min)** | **Target ion** | **Q1 Ion** | **Relative response (%)** | **R^2^** | **Range (μg)** |
| --- | --- | --- | --- | --- | --- | --- | --- |
| Proline | Cyclo-leucine | 8.24 | 216.1 | 147.1 | 91.2 | 0.992 | 10-200 |
| Malate | Castanospermine | 10.63 | 335.1 | 265.1 | 61.2 | 0.995 | 10-200 |
| Glucose | Cyclo-leucine | 15.14 | 364.2 | 291.1 | 197.8 | 0.986 | 1 -50 |
| Fructose | Cyclo-leucine | 14.88 | 364.2 | 307.2 | 801.6 | 0.979 | 1 -50 |
| *Myo-*inositol | Cyclo-leucine | 17.08 | 432.3 | 265.1 | 176.2 | 0.992 | 1 -50 |
| Pinitol | Castanospermine | 14.47 | 433.3 | 318.2 | 458.8 | 0.996 | 1 -50 |
| Pinitol | Castanospermine | 14.47 | 433.2 | 374.2 | 108.9 | 0.977 | 50-200 |
| Pinitol | Castanospermine | 14.47 | 462.2 | 387.2 | 318.5 | 0.945 | 200-500 |
| Castanospermine* | * | 16.62 | 462.3 | 387.2 | 369.2 | * | * |
| Cyclo-leucine* | * | 9.17 | 230.1 | 168.0 | 51.5 | * | * |
